# WyNDA: A method to discover mathematical models of dynamical systems from data

**DOI:** 10.1016/j.mex.2024.102625

**Published:** 2024-02-21

**Authors:** Agus Hasan

**Affiliations:** Department of ICT and Natural Sciences, Norwegian University of Science and Technology, Larsgårdsvegen 2, Ålesund, 6009, Norway

**Keywords:** Dynamical systems, Data-driven method, Governing equations, Wide-Array of Nonlinear Dynamics Approximation

## Abstract

This paper introduces a novel method called Wide-Array of Nonlinear Dynamics Approximation (WyNDA) for extracting mathematical models of dynamical systems from data. A key advantage of this method over existing approaches lies in its suitability for online implementation. Moreover, WyNDA stands out by not relying on optimization or machine learning, ensuring computational efficiency. The fundamental concept revolves around approximating the unknown function of a dynamical system through a diverse set of basis functions that encapsulate the available data. An adaptive observer is then employed to iteratively refine this approximation and estimate the associated parameters. The efficacy of the proposed method is demonstrated through numerical simulations encompassing linear systems, nonlinear systems, and control systems. The results underscore the method’s ability to successfully unveil the governing equations of dynamical systems, highlighting its potential for extracting intricate system dynamics from observational data.

• WyNDA represents a novel approach for uncovering mathematical models of dynamical systems from data.
• Utilizing a series of basis functions, WyNDA effectively approximates the unknown structure inherent in dynamical systems.
• The validation of WyNDA involves benchmark equations of dynamical systems, confirming its efficacy in diverse scenarios.

## Graphical abstract

**Figure:**
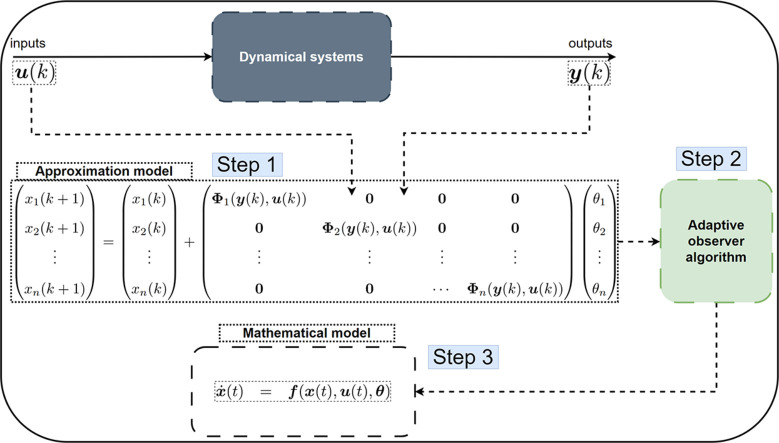

Specification table

**Table:** 

Subject area:	Mathematics
More specific subject area:	Dynamical Systems
Name of your method:	Wide-Array of Nonlinear Dynamics Approximation
Name and reference of original method:	Not applicable
Resource availability:	https://github.com/agushasan/discovery

## Method details

A dynamical system refers to a system in which its state undergoes changes over time and follows a predetermined set of rules or equations [1]. The mathematical models of dynamical systems capture the behavior and changes of variables within a system, providing a framework to study and predict its dynamics. Dynamical systems can be either discrete or continuous. In discrete dynamical systems, time advances in distinct steps, while continuous dynamical systems involve a continuous and smooth evolution over time. The state variables of a dynamical system represent the system’s internal configuration, and the equations governing their changes describe how the system evolves. The study of dynamical systems is crucial in various fields, including physics, engineering, biology, economics, and ecology. It enables researchers to analyze and understand the behavior of complex systems, predict their future states, and design control strategies for desired outcomes [2], [3], [4], [5], [6].

**Fig. 1:**
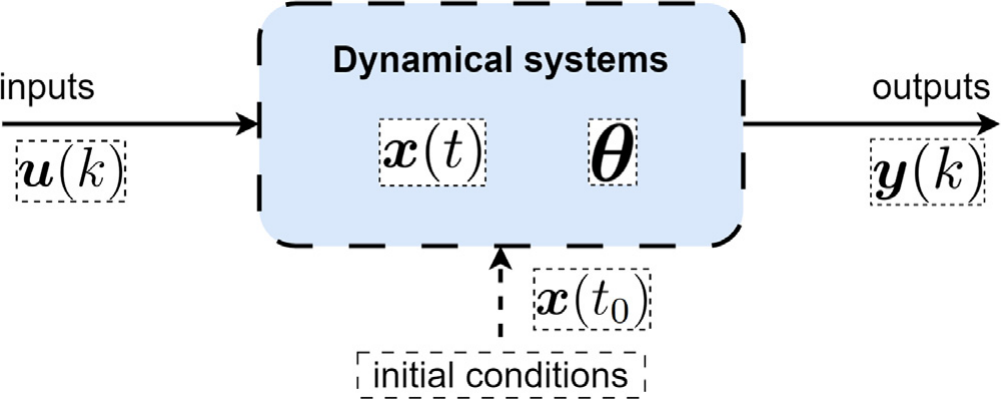
A schematic depiction of the input-output relationship in dynamical systems.

The schematic diagram in Fig. 1 illustrates a model of a dynamical system with its input and output. This system is characterized by a state vector, denoted as $x\in R^{n}$, which is a function of the time variable t. The state vector represents the internal variables that describe the system’s behavior over time. Additionally, the system incorporates a parameter vector $\theta \in R^{r}$, introducing a distinguishing factor among different instances of the system. Moreover, the dynamical system is characterized by an initial condition, denoted as $x(t_{0})$, representing the state of the system at the initial time, providing a starting point for its evolution. The inclusion of initial conditions is crucial for capturing the system’s history and understanding its trajectory from a specified starting state. The system is responsive to control inputs, denoted by $u\in R^{p}$, which influence its behavior. The control input serves as an external factor that can be manipulated to achieve desired outcomes or responses from the system. This feature makes dynamical systems amenable to control strategies, where inputs can be adjusted to attain specific performance objectives.

As the system evolves, it produces an output that is recorded through a sensor system. The output, denoted as y(k), is measured in discrete-time steps indexed by k. Note that, throughout the remainder of the paper, the index k is adopted to denote discrete-time instances, representing instances in a digital or sampled domain. Conversely, the symbol t is utilized to signify continuous-time. Hence, x(k) designates the discrete-time state variable, while x(t) denotes the continuous-time state variable. Discrete-time measurements are common in practical applications, reflecting the sampling nature of sensor systems. The recorded output provides information about the system’s state, allowing for monitoring, analysis, and control adjustments. Understanding the dynamics of such systems is essential for various applications, including engineering, physics, biology, and economics. The schematic diagram encapsulates the key components of a dynamical system, emphasizing the interactions between the state, parameters, initial conditions, control inputs, and sensor-recorded outputs. This holistic representation forms the basis for modeling and analyzing the behavior of dynamic systems in diverse fields.

Developing mathematical models of dynamical systems involves two primary approaches: deriving models from first principles using the laws of physics (mechanistic models) or constructing models from sensor data through optimization or machine learning techniques [7]. Each approach has its strengths and challenges, catering to different preferences and requirements. The first approach relies on a deep understanding of the underlying physics governing the system. This involves formulating equations based on fundamental principles and laws. While this method provides models that are rooted in physical reality, it demands a strong background in physics and a comprehensive knowledge of the system’s dynamics. The derived models are often complex and require precise parameter tuning, making them suitable for well-understood systems where the governing equations are known [8], [9], [10], [11], [12], [13], [14]. On the other hand, machine learning techniques, particularly those involving data-driven approaches, offer an alternative route to model development. This approach utilizes sensor data measurements to construct models without a prior knowledge of the system’s governing equations. Machine learning algorithms, such as neural networks or support vector machines, learn patterns and relationships from the data to generate predictive models [15], [16], [17], [18], [19], [20], [21], [22]. While this method is versatile and applicable to a wide range of systems, it often produces black-box models, making it challenging to interpret the underlying dynamics. However, regardless of the approach chosen, the validation of dynamical models is a critical step. Models derived from either first principles or data-driven methods must be tested against real-world data to ensure their accuracy and reliability.

There is a growing incentive to discover mathematical models based on data that not only exhibit predictive power but also offer interpretability [23], [24], [25], [26], [27]. Explainable models are advantageous as they provide insights into the underlying mechanisms of the system, enhancing understanding of its behavior. Achieving a balance between accuracy and interpretability is a key challenge in this field, as it requires methods that can capture complex dynamics while remaining transparent and comprehensible to domain experts. The exploration of deriving governing equations from data has been a focal point within the research community over the past decades. One notable instance is found in [28], where the authors employ sparse identification of nonlinear dynamical systems to discern the equations governing a dynamic system based on empirical data. The effectiveness of the proposed method, known as Sparse Identification of Nonlinear Dynamics (SINDy), is contingent upon factors such as the selection of measurement variables, the quality of the data, and the choice of the sparsifying basis function. The method’s success hinges on these considerations, making it crucial to carefully tailor these aspects to ensure accurate and meaningful identification of the underlying dynamics. In a more recent study [29], the authors introduced Automatic Regression for Governing Equations (ARGOS), a novel approach that seamlessly integrates machine learning and statistical inference to automatically discern interpretable models characterizing the dynamics of a system. The algorithm encompasses crucial phases such as data smoothing and numerical approximation of derivatives, incorporating bootstrap sampling along with sparse regression to establish confidence intervals for variable selection. Notably, the method demonstrates its capability to consistently identify three-dimensional systems when provided with moderately-sized time series data and high signal quality in relation to background noise levels.

The references mentioned earlier are undeniably robust, yet their efficacy lies predominantly based on optimization methods or machine learning, requiring data to be collected before processing. In contrast, the proposed method introduces three innovative features. Firstly, it enables online implementation, allowing real-time processing without the need for a pre-existing dataset. This real-time adaptability is particularly advantageous in dynamic scenarios where prompt decision-making is essential. Secondly, the proposed method leverages an adaptive observer, which dynamically approximates the underlying dynamical systems based on their convergence rate. This adaptive nature enhances the model’s accuracy and adaptability to changing conditions. Thirdly, the method exhibits efficiency by requiring fewer data points for highly excited systems, as evidenced by numerical examples. This reduction in data demand not only streamlines the implementation process but also makes the approach more resource-efficient. Together, these features position the proposed method as a versatile and efficient solution, well-suited for dynamic systems where real-time adaptability and resource optimization are paramount. Given its broad applicability across various dynamical systems and the use of wide-array of basis functions, the proposed methodology is coined as Wide-Array of Nonlinear Dynamics Approximation or WyNDA, as detailed in the next section.

In mathematical terms, the continuous-time dynamical system is described by the following equations:

(1) $$\dot{x}\left(t\right)=f\left(x\left(t\right),u\left(t\right),\theta \right)$$

(2) $$x\left(t_{0}\right)=x_{0}$$

These equations capture the evolution of the system’s state over time. The first equation, a vector differential equation, expresses the rate of change of the state vector x(t) with respect to time. This rate of change is determined by a function f that takes into account the current state x(t), control input u(t), and parameters θ. The function f encapsulates the underlying dynamics governing the system’s behavior. The second equation sets the initial condition for the system. At the initial time $t=t_{0}$, the state vector $x(t_{0})$ is specified as $x_{0}$.

In many scientific and engineering applications, the functions f and the parameters θ governing a dynamical system are often unknown. However, the state vector x(t) can be measured using sensors, and the inputs u(t) are typically known as they are intentionally applied to the system. The fundamental challenge in discovering the mathematical model of a dynamical system lies in identifying the unknown function f and its associated parameters θ from data. This process is crucial for understanding and predicting the behavior of the system. By deciphering the underlying dynamics encoded in f and determining the specific values of θ, researchers and engineers gain valuable insights into how the system responds to inputs and evolves over time. The ability to accurately capture these dynamics enhances the overall comprehension of complex systems, enabling informed decision-making and control strategies. The primary approach to discover the mathematical models involves leveraging measured data, particularly the recorded measurement vector y(k) obtained from sensors, which can be modelled as follows:

(3) y(k)=x(k)+v(k)

The term v(k)∼N(0,R(k)) is employed to characterize the presence of noise or uncertainty in the sensor data. This additional term allows for the consideration of potential inaccuracies in the measurements, contributing to a more robust and realistic modeling of the system.

The objective of this paper is to discover mathematical models of dynamical systems represented by Eq. (1) that captures the system’s dynamics based on data from sensor measurement (3). This is referred to as an inverse problem, constituting one of the most crucial topics in modeling and simulation. The problem is particularly relevant in scenarios where a detailed understanding of the underlying processes is essential, such as in the design and optimization of control systems, robotics, and many other applications across various scientific and engineering domains.

## Wide-array of nonlinear dynamics approximation (WyNDA)

**Fig. 2:**
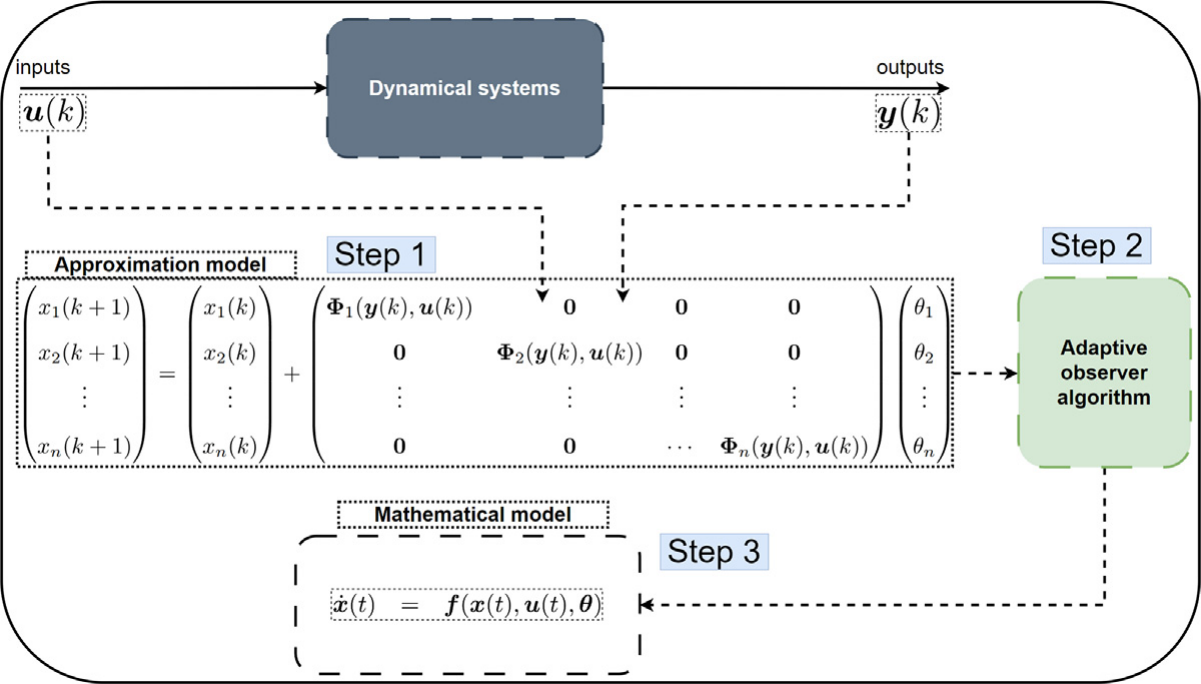
A schematic diagram of the proposed methodology based on Wide-Array of Nonlinear Dynamics Approximation (WyNDA) to discover mathematical models of dynamical systems from data.

The proposed method for discovering the mathematical model of dynamical systems represented by (1) is called WyNDA and involves several key steps, as outlined in Fig. 2.

• *The first step* is to construct an approximation model based on wide-array of basis functions that captures and stores the measurement data y(k) generated by the dynamical system and control input u(k). This approximation model, built from the observed data, serves as a basis for further analysis.
• *The second step* is the application of an adaptive observer algorithm. This algorithm is instrumental in estimating the system’s parameter θ. The adaptive observer iteratively refines its estimation of θ by comparing the predicted and measured outputs, thereby enhancing the accuracy of the parameter estimation.
• *The third step* is to convert the discrete-time approximation model into continuous-time model. In this case, once a sufficiently accurate estimate $\bar{\theta }$ is obtained, it enables the construction of the mathematical model of the dynamical system (1).

The discovered model, now incorporating the estimated parameters, provides a mathematical representation of the underlying dynamics. This representation is then capable of predicting the system’s response to different inputs with increased fidelity. This methodology offers a systematic and data-driven approach to modeling dynamical systems. By leveraging measured data, the approach avoids the need for a prior knowledge of the system dynamics, making it applicable in situations where the underlying governing equations are unknown or complex. The adaptive observer plays a crucial role in refining the model, ensuring that the estimated parameters align closely with the true characteristics of the dynamical system. The resulting model, grounded in observed data, serves as a valuable tool for understanding, predicting, and controlling the system’s behavior.

To this end, the approximation model is expressed as follows:

(4) $$\begin{array}{c} x(k+1)=x(k)+\Psi (y(k),u(k))\theta \end{array}$$

where $\Psi \left(y\left(k\right),u\left(k\right)\right)\in R^{n\times r}$ is called the approximation function. Note that, for any dynamical system represented by (1), its Euler discretization yields:

(5) $$\begin{array}{c} x(k+1)=x(k)+\Delta tf(x(k),u(k),\theta ) \end{array}$$

Thus, the mathematical model of the dynamical system can be approximated by the approximation model by comparing (4) and (5), as follows:

(6) $$\begin{array}{c} \Delta tf(x(k),u(k),\theta )\approx \Psi (y(k),u(k))\theta \end{array}$$

The approximation function Ψ(y(k),u(k)) consists of a wide-array of basis functions and is given by:

(7) $$\begin{array}{ccc} \Psi (y(k),u(k)) & = & \left(\begin{array}{cccc} \Phi_{1}\left(y\left(k\right),u\left(k\right)\right) & 0 & 0 & 0\\ 0 & \Phi_{2}\left(y\left(k\right),u\left(k\right)\right) & 0 & 0\\ \vdots & \vdots & \vdots & \vdots \\ 0 & 0 & \cdots & \Phi_{n}\left(y\left(k\right),u\left(k\right)\right) \end{array}\right) \end{array}$$

The array of basis functions $\Phi_{j}\left(y\left(k\right),u\left(k\right)\right)$, where j=1,⋯,n, is not unique and can be chosen in various ways. An example is given as follows:

(8) $$\begin{array}{ccc} \Phi_{j}\left(y\left(k\right),u\left(k\right)\right) & = & \left(\begin{array}{cccccccc} 1 & y_{i}\left(k\right) & \cdots & y_{i}^{2}\left(k\right) & \cdots & \sin(y_{i}\left(k\right)) & \cdots & \cos(y_{i}\left(k\right)) \end{array}\right) \end{array}$$

In this case, the basis function comprises a constant, polynomials, and trigonometric functions. These elements collectively form a set of functions used for approximating the unknown function f in the dynamical system. The inclusion of a constant term, polynomial functions, and trigonometric functions allows for a flexible and diverse representation of the system’s dynamics, capturing various patterns and behaviors that may be present in the underlying function. The choice of these functions is intuitive, as polynomials and trigonometric functions are frequently employed in function approximation, such as in Taylor and Fourier series. However, other combinations of these functions are also possible. For instance, including $|y_{l}\left(k\right)|y_{i}{\left(k\right)}^{3}$ or $y_{l}\left(k\right)\sin\left(y_{i}\left(k\right)\right)$ in the set of basis functions is a viable option. Once the approximation model with its basis functions is constructed, the subsequent step involves applying an estimation algorithm to determine the parameter vector θ in (4). This estimation process is crucial for obtaining the values of θ that best align with the underlying dynamics of the dynamical system, ensuring an accurate representation of the system’s behavior.

The estimation algorithm proposed in this paper relies on an adaptive observer presented in [30]. This method exhibits notable strengths, particularly its capacity to rapidly converge to the actual parameter values. Adaptive observers are advantageous for their ability to adapt and fine-tune the estimated parameters based on real-time data. In the context of dynamical systems, this adaptability is crucial for capturing the system’s intricate dynamics, providing a more accurate representation. The observer continuously refines its estimation, ensuring that the identified parameter values closely align with the true system characteristics. This adaptability contributes to the overall effectiveness of the estimation algorithm, making it a robust tool for discovering the underlying parameters governing the dynamical system. The adaptive observer to estimate the approximation model (4) is designed as follow:

(9) $$\bar{x}\left(k|k\right)=\bar{x}\left(k|k-1\right)+\left(K_{x}\left(k\right)+\Gamma \left(k|k\right)K_{\theta }\left(k\right)\right)\left(y\left(k\right)-\bar{x}\left(k|k-1\right)\right)$$

(10) $$\bar{\theta }\left(k|k\right)=\bar{\theta }\left(k|k-1\right)-K_{\theta }\left(k\right)\left(y\left(k\right)-\bar{x}\left(k|k-1\right)\right)$$

where the observer gains $K_{x}\left(k\right)\in R^{n\times n}$, $K_{\theta }\left(k\right)\in R^{r\times n}$, and $\Gamma \left(k|k\right)\in R^{n\times r}$ are calculated from the following formulas:

(11) $$K_{x}\left(k\right)=P_{x}\left(k|k-1\right){\left(P_{x}\left(k|k-1\right)+R_{x}\left(k\right)\right)}^{-1}$$

(12) $$\Omega \left(k\right)=\Gamma \left(k|k-1\right)P_{\theta }\left(k|k-1\right)\Gamma^{⊺}\left(k|k-1\right)+R_{\theta }\left(k\right)$$

(13) $$K_{\theta }\left(k\right)=P_{\theta }\left(k|k-1\right)\Gamma^{⊺}\left(k|k-1\right)\Omega {\left(k\right)}^{-1}$$

(14) $$\Gamma \left(k|k\right)=\left(I-K_{x}\left(k\right)\right)\Gamma \left(k|k-1\right)$$

The observer gains depend on the update matrices $P_{x}\left(k|k-1\right)\in R^{n\times n}$ and $P_{\theta }\left(k|k-1\right)\in R^{r\times r}$ and the tuning matrices $R_{x}\left(k\right)\in R^{n\times n}$ and $R_{\theta }\left(k\right)\in R^{n\times n}$. The initial conditions for this matrices are $P_{x}\left(0|-1\right)={\left(P_{x}\left(0|-1\right)\right)}^{⊺}>0$, $P_{\theta }\left(0|-1\right)={\left(P_{\theta }\left(0|-1\right)\right)}^{⊺}>0$, $R_{x}\left(1\right)={\left(R_{x}\left(1\right)\right)}^{⊺}>0$, $R_{\theta }\left(1\right)={\left(R_{\theta }\left(1\right)\right)}^{⊺}>0$, and $\Gamma {\left(0|-1\right)}^{⊺}\Gamma \left(0|-1\right)>0$.

The prediction updates are given by:

(15) $$\bar{x}\left(k+1|k\right)=\bar{x}\left(k|k\right)+\Psi \left(y\left(k\right),u\left(k\right)\right)\bar{\theta }\left(k|k\right)$$

(16) $$\bar{\theta }\left(k+1|k\right)=\bar{\theta }\left(k|k\right)$$

(17) $$P_{x}\left(k+1|k\right)=\lambda_{x}^{-1}\left(I-K_{x}\left(k\right)\right)P_{x}\left(k|k-1\right)$$

(18) $$P_{\theta }\left(k+1|k\right)=\lambda_{\theta }^{-1}\left(I-K_{\theta }\left(k\right)\Gamma \left(k|k-1\right)\right)P_{\theta }\left(k|k-1\right)$$

(19) Γ(k+1|k)=Γ(k|k)−Ψ(y(k),u(k))

Eq. (15) articulates the structure of the approximation model, serving as a representation of the dynamical system under consideration. This model captures the system’s behavior and dynamics, providing an insightful mathematical framework. On the other hand, Eq. (16) underscores a critical characteristic of the parameter θ; it is considered constant or piece-wise constant. This implies that the parameter remains fixed within certain intervals or segments, reflecting the system’s behavior during those specific periods. The constancy or piece-wise constancy of θ is a key assumption in the proposed methodology, enabling a more tractable approach to parameter estimation. This assumption aligns with the notion that certain system characteristics, represented by θ, may remain relatively stable or undergo discernible changes over distinct phases of the system’s operation. Therefore, Eqs. (15) and (16) collectively form the basis for the parameter estimation algorithm, offering a structured and nuanced understanding of the dynamical system’s representation and the constancy characteristics of the parameter of interest. The prediction update has two tuning parameters $\lambda_{x},\lambda_{\theta }\in \left(0,1\right)$, which can be tuned to increase the performance of the estimator.

The prerequisite for the convergence of the parameter estimate is that the sequence Ψ(k) must exhibit persistent excitation. Under this circumstance, there exist fixed positive constants κ and ξ such that:

(20) $$\begin{array}{c} 0<\kappa I\leq \Sigma_{l=k-\xi }^{k}\Psi {\left(l\right)}^{⊺}\Psi \left(l\right) \end{array}$$

Indeed, the persistence of excitation is a fundamental and indispensable factor in the context of parameter estimation. In the area of system identification or modeling, persistency of excitation denotes the continuous and sufficient variation in the input signals or stimuli, ensuring that the system under consideration remains responsive and informative for the duration of the estimation process. Subject to the persistence of excitation condition (20), we can ensure the convergence of the estimate to the true value, as asserted in the following theorem.

Theorem 1 *The estimation errors:*

(21) $$\begin{array}{ccc} \epsilon_{x}\left(k\right) & = & \bar{x}\left(k|k\right)-x\left(k\right) \end{array}$$

(22) $$\begin{array}{ccc} \epsilon_{\theta }\left(k\right) & = & \bar{\theta }\left(k|k\right)-\theta \end{array}$$

*exhibit exponential decay towards zero, and the convergence speed can be adjusted arbitrarily by tuning the parameters*$\lambda_{x}$*and*$\lambda_{\theta }$*.*

Proof Building upon the concept introduced in [30], [31], and [32], we will initially establish the exponential convergence of a specific linearly combined error. In this context, the error is defined as:

(23) $$\begin{array}{c} \epsilon \left(k\right)=\epsilon_{x}\left(k\right)+\Gamma \left(k|k\right)\epsilon_{\theta }\left(k\right) \end{array}$$

First remark that, substituting (10) to (9), yields:

(24) $$\begin{array}{ccc} \bar{x}\left(k|k\right) & = & \bar{x}\left(k|k-1\right)+K_{x}\left(k\right)\left(x\left(k\right)-\bar{x}\left(k|k-1\right)\right)\\ & & -\;\Gamma \left(k|k\right)\left(\bar{\theta }\left(k|k\right)-\bar{\theta }\left(k|k-1\right)\right) \end{array}$$

The first term of the right hand side of (24) can be obtained from (15), as follows:

(25) $$\begin{array}{ccc} \bar{x}\left(k|k-1\right) & = & \bar{x}\left(k-1|k-1\right)+\Psi \left(y\left(k-1\right),u\left(k-1\right)\right)\bar{\theta }\left(k-1|k-1\right) \end{array}$$

Note that from (4) and (21), we have:

(26) $$\begin{array}{c} x\left(k\right)=\bar{x}\left(k-1|k-1\right)-\epsilon_{x}\left(k-1\right)+\Psi \left(y\left(k-1\right),u\left(k-1\right)\right)\theta \end{array}$$

Thus, utilizing (22), the second term of the right hand side of (24) is given by:

(27) $$\begin{array}{ccc} K_{x}\left(k\right)\left(x\left(k\right)-\bar{x}\left(k|k-1\right)\right) & = & -K_{x}\left(k\right)\epsilon_{x}\left(k-1\right)\\ & & -\;K_{x}\left(k\right)\Psi \left(y\left(k-1\right),u\left(k-1\right)\right)\epsilon_{\theta }\left(k-1\right) \end{array}$$

The third term of the right hand side of (24) is given by:

(28) $$\begin{array}{ccc} -\Gamma \left(k|k\right)\left(\bar{\theta }\left(k|k\right)-\bar{\theta }\left(k|k-1\right)\right) & = & -\Gamma \left(k|k\right)\left(\epsilon_{\theta }\left(k\right)-\epsilon_{\theta }\left(k-1\right)\right) \end{array}$$

Combining (25), (27), and (28), (24) can be written as:

(29) $$\begin{array}{ccc} \bar{x}\left(k|k\right) & = & \bar{x}\left(k-1|k-1\right)+\Psi \left(y\left(k-1\right),u\left(k-1\right)\right)\bar{\theta }\left(k-1|k-1\right)\\ & & -\;K_{x}\left(k\right)\epsilon_{x}\left(k-1\right)-K_{x}\left(k\right)\Psi \left(y\left(k-1\right),u\left(k-1\right)\right)\epsilon_{\theta }\left(k-1\right)\\ & & -\;\Gamma \left(k|k\right)\left(\epsilon_{\theta }\left(k\right)-\epsilon_{\theta }\left(k-1\right)\right) \end{array}$$

From (21), we have:

(30) $$\begin{array}{ccc} \epsilon_{x}\left(k+1\right) & = & \bar{x}\left(k+1|k+1\right)-x\left(k+1\right) \end{array}$$

Substituting (4) and (29) into (30), we have:

(31) $$\begin{array}{ccc} \epsilon_{x}\left(k+1\right) & = & \left(I-K_{x}\left(k+1\right)\right)\epsilon_{x}\left(k\right)+\left(I-K_{x}\left(k+1\right)\right)\Psi \left(y\left(k\right),u\left(k\right)\right)\epsilon_{\theta }\left(k\right)\\ & & -\;\Gamma \left(k+1|k+1\right)\left(\epsilon_{\theta }\left(k+1\right)-\epsilon_{\theta }\left(k\right)\right) \end{array}$$

Note that from (14) and (19), we have:

(32) $$\begin{array}{ccc} \Gamma (k+1|k+1) & = & \left(I-K_{x}\left(k+1\right)\right)\left(\Gamma (k|k)-\Psi (y(k),u(k))\right) \end{array}$$

Thus, from (23) we obtain:

(33) $$\begin{array}{ccc} \epsilon (k+1) & = & \epsilon_{x}\left(k+1\right)+\Gamma \left(k+1|k+1\right)\epsilon_{\theta }\left(k+1\right)\\ & = & \left(I-K_{x}\left(k+1\right)\right)\epsilon \left(k\right) \end{array}$$

The expression (33) aligns with the dynamics of the estimation error in a design employing an exponential forgetting factor derived in [33] for homogeneous systems:

(34) x(k+1)=x(k)

(35) y(k)=x(k)

Consequently, the sequence ϵ(k+1) exhibits exponential decay towards zero. Considering Eq. (23), to ensure the exponential convergence of the observer, it suffices to verify that $\epsilon_{\theta }\left(k\right)$ also undergoes exponential decay. This is demonstrated sequentially as follows:

(36) $$\begin{array}{c} \epsilon_{\theta }\left(k+1\right)=\bar{\theta }\left(k|k\right)-K_{\theta }\left(k+1\right)\left(x\left(k+1\right)-\bar{x}\left(k+1|k\right)\right)-\theta \end{array}$$

Substituting (21) and (22) into (36), we have:

(37) $$\begin{array}{c} \epsilon_{\theta }\left(k+1\right)=\epsilon_{\theta }\left(k\right)+K_{\theta }\left(k+1\right)\epsilon_{x}\left(k\right)+K_{\theta }\left(k+1\right)\Psi \left(y\left(k\right),u\left(k\right)\right)\epsilon_{\theta }\left(k\right) \end{array}$$

Finally, substituting (23) and (19) into (37), we have:

(38) $$\begin{array}{c} \epsilon_{\theta }\left(k+1\right)=\left(I-K_{\theta }\left(k+1\right)\Gamma \left(k+1|k\right)\right)\epsilon_{\theta }\left(k\right)+K_{\theta }\left(k+1\right)\epsilon \left(k\right) \end{array}$$

This error corresponds to the dynamics of the estimation error in a system designed with an exponential forgetting factor presented in [30], as illustrated below:

(39) $$\begin{array}{ccc} \theta (k+1) & = & \theta (k) \end{array}$$

(40) $$\begin{array}{ccc} y_{\theta }\left(k\right) & = & \Gamma (k|k-1)\theta (k) \end{array}$$

Hence, the error $\epsilon_{\theta }\left(k\right)$ undergoes exponential decay towards zero. Given the relationship in (23), this implies that the estimation error $\epsilon_{x}\left(k\right)$ also experiences exponential decay towards zero. This concludes the proof. □

Having the convergence of the estimate is guaranteed, as outlined in Theorem 1, we can now provide a summary of the WyNDA algorithm, which is presented in Algorithm 1. This algorithm encapsulates the key steps and procedures for the efficient functioning of the WyNDA methodology.

**Algorithm 1:**
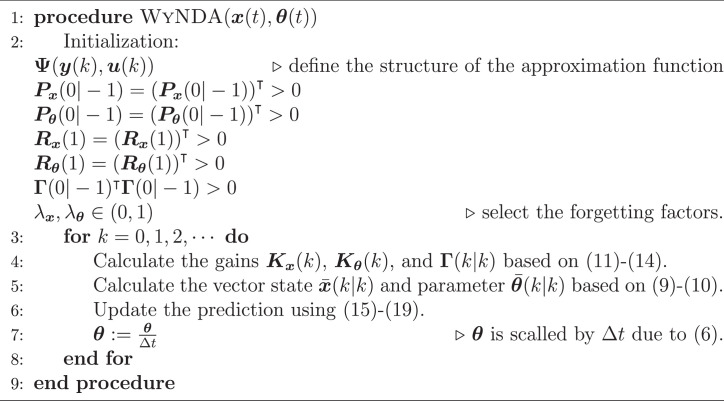
Wide-Array of Nonlinear Dynamics Approximation (WyNDA)

## Method validation

In this section, four examples are presented to validate the effectiveness of WyNDA. The first example demonstrates the application of WyNDA to discover a mathematical model of a linear system from data. Subsequently, the second example showcases the method’s capability to estimate nonlinear systems. Here, four nonlinear mathematical equations are considered: the Lorenz system, Rössler attractor, Lotka–Volterra equations, and Van der Pol oscillator. Moving forward, the third example illustrates how the proposed method can be effectively employed for control system applications. Lastly, the fourth example demonstrates the utility of the method in the context of system identification. These diverse examples collectively highlight the versatility and robustness of WyNDA across various types of dynamical systems and applications. All codes and data are available in: https://github.com/agushasan/discovery.

### Example 1: linear systems

**Fig. 3:**
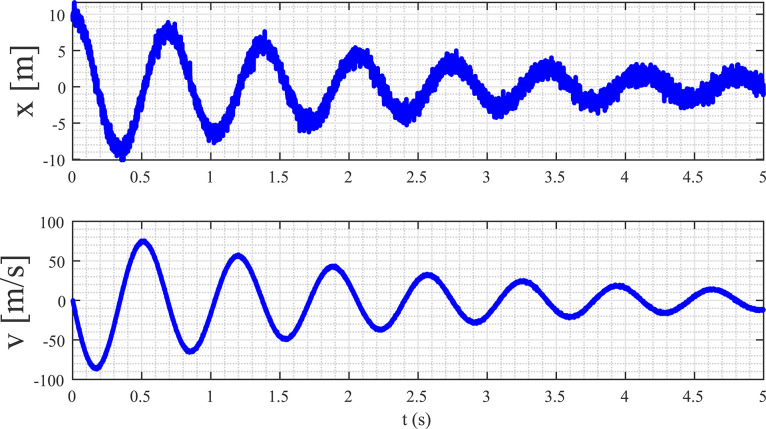
Captured measurements of the MSD system’s position and velocity data from Matlab Simulink simulation.

The primary aim of this example is to derive a mathematical model for a mass-spring-damper (MSD) system. This system is characterized by a first-order linear differential equation encompassing two state variables: position (x) and velocity (v). Notably, the MSD system is defined by three key parameters, namely the mass (m), the spring coefficient (k), and the damper coefficient (b). To initiate this process, we conduct a simulation of MSD system utilizing Matlab Simulink to gather pertinent data with m=1 kg, k=84 N/m, and b=0.9 NS/m. The data is sampled at a rate of 1 ms, and to introduce uncertainty in the measurements, a white Gaussian noise with a standard deviation of 1 is added. The resulting data is systematically compiled and stored in a file named DATAMSD.mat. A visual representation of this data is presented in Fig. 3, offering a graphical insight into the dynamics of the system. This dataset will serve as the foundation for subsequent analysis and the formulation of an accurate mathematical model for the MSD system.

In this example, the basis function is selected as follow:

(41) $$\begin{array}{c} \Phi_{j}\left(y\left(k\right),u\left(k\right)\right)={\left(\begin{array}{c} y_{1}\left(k\right)\\ y_{2}\left(k\right)\\ y_{1}^{2}\left(k\right)\\ y_{2}^{2}\left(k\right)\\ y_{1}\left(k\right)y_{2}\left(k\right) \end{array}\right)}^{⊺} \end{array}$$

where $y_{1}\left(k\right)$ represents the measurement of x(k) and $y_{2}\left(k\right)$ represents the measurement of v(k). Thus, the approximation model is given by:

(42) $$\begin{array}{ccc} & & \left(\begin{array}{c} x(k+1)\\ v(k+1) \end{array}\right)=\left(\begin{array}{c} x(k)\\ v(k) \end{array}\right)+{\left(\begin{array}{cc} y_{1}\left(k\right) & 0_{5\times 1}\\ y_{2}\left(k\right) & y_{1}\left(k\right)\\ y_{1}^{2}\left(k\right) & y_{2}\left(k\right)\\ y_{2}^{2}\left(k\right) & y_{1}^{2}\left(k\right)\\ y_{1}\left(k\right)y_{2}\left(k\right) & y_{2}^{2}\left(k\right)\\ 0_{5\times 1} & y_{1}\left(k\right)y_{2}\left(k\right) \end{array}\right)}^{⊺}\left(\begin{array}{c} \theta_{1}\\ \vdots \\ \theta_{10} \end{array}\right) \end{array}$$

Since the MSD system is linear, we would expect parameters correspond to the nonlinear terms in (42) (such as $\theta_{3}$, $\theta_{4}$, $\theta_{5}$, $\theta_{8}$, $\theta_{9}$, $\theta_{10}$) are zero. The subsequent step involves implementing the adaptive observer (9)-(10) to obtain the state estimate $\bar{x}$ and parameter estimate $\bar{\theta }$. The parameters for the adaptive algorithm are given in Table 1.

**Table 1:** Values of parameters used in simulation of example 1.

parameter	value	parameter	value
$P_{x}\left(0|-1\right)$	0.1I	$\lambda_{x}$	0.995
$P_{\theta }\left(0|-1\right)$	0.1I	$\lambda_{\theta }$	0.999
$R_{x}\left(1\right)$	I	Γ(0|0)	0
$R_{\theta }\left(1\right)$	I		

**Fig. 4:**
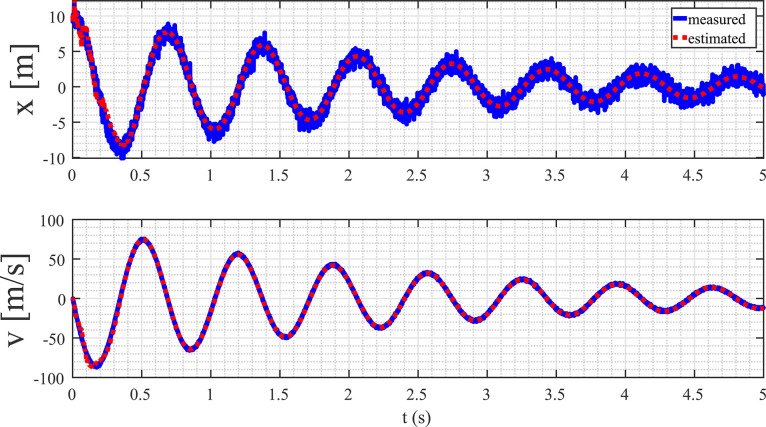
State estimation from measured data.

**Fig. 5:**
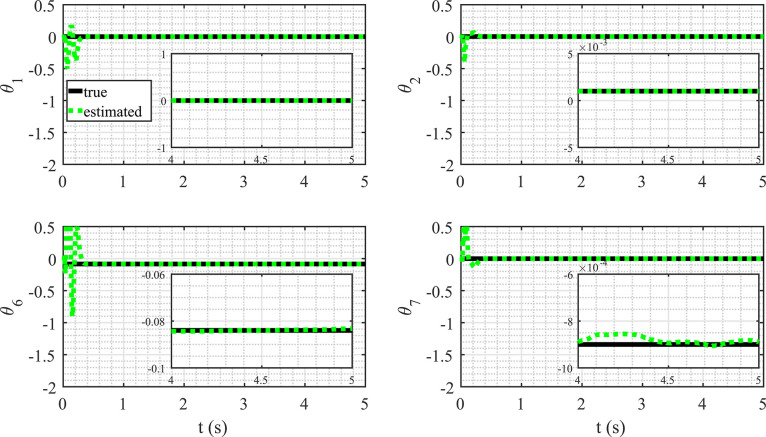
Estimated parameters for the MDS system.

The outcomes are showcased in Figs. 4 and 5, respectively. Analyzing Fig. 4, it is evident that the algorithm successfully mitigates the impact of noise, significantly improving the data quality for both variables. To assess the effectiveness of the proposed method, we analyze the convergence of the estimated values towards the actual values. Convergence, in this context, is gauged by scrutinizing the trend and alignment between the estimated and actual values over successive iterations or time intervals. This evaluation provides insights into how well the proposed method approximates the true underlying values of the system dynamics. Fig. 5 displays the evolution of the parameter estimate, illustrating its convergence toward the actual values. From the depicted figure, it is evident that the algorithm converges to the true values within a time span of 0.4 s, corresponding to 400 data points. This observation underscores the efficiency and rapid convergence of the algorithm in accurately approximating the sought-after values. The temporal aspect, represented by the 0.4-second interval, provides insights into the algorithm’s convergence speed and its ability to converge within a relatively short duration.

Table 2 presents the estimated parameters for the approximation model (42). As anticipated, the parameters linked to nonlinear basis functions are determined to be zero. The corresponding approximation model (42) for the MSD system is expressed as:

(43) $$\begin{array}{ccc} & & \left(\begin{array}{c} x(k+1)\\ v(k+1) \end{array}\right)=\left(\begin{array}{c} x(k)\\ v(k) \end{array}\right)+\left(\begin{array}{cc} 0 & 0.001\\ -0.083 & -0.001 \end{array}\right)\left(\begin{array}{c} x(k)\\ v(k) \end{array}\right) \end{array}$$

Since the sampling time Δt is 1 ms, the continuous time differential equation for the MSD system is given by:

(44) $$\begin{array}{ccc} \left(\begin{array}{c} \dot{x}\left(t\right)\\ \dot{v}\left(t\right) \end{array}\right) & = & \left(\begin{array}{cc} 0 & 1\\ -83 & -1 \end{array}\right)\left(\begin{array}{c} x(t)\\ v(t) \end{array}\right) \end{array}$$

This outcome corresponds to parameters m=1 kg, k=83 N/m, and b=1 NS/m, which slightly deviate from the Matlab Simulink simulation data where parameters were set to m=1 kg, k=84 N/m, and b=0.9 NS/m. The observed discrepancy can be attributed to noise introduced during the Simulink simulation.

**Table 2:** Comparison between the true and estimated parameters in the MSD system.

parameter	estimated value	parameter	estimated value
$\theta_{1}$	0	$\theta_{6}$	-0.083
$\theta_{2}$	0.001	$\theta_{7}$	-0.001
$\theta_{3}$	0	$\theta_{8}$	0
$\theta_{4}$	0	$\theta_{9}$	0
$\theta_{5}$	0	$\theta_{10}$	0

### Example 2: nonlinear systems

The objective of this example is to demonstrate the performance of the proposed method for nonlinear systems. Specifically, four nonlinear mathematical equations are examined: the Lorenz system, Rössler attractor, Lotka–Volterra equations, and Van der Pol oscillator. Note that in these examples, the time series are expressed in units of seconds. The Lorenz system is given by:

(45) $$\dot{p}\left(t\right)=\sigma \left(q\left(t\right)-p\left(t\right)\right)$$

(46) $$\dot{q}\left(t\right)=p\left(t\right)\left(\rho -r\left(t\right)\right)-q\left(t\right)$$

(47) $$\dot{r}\left(t\right)=p\left(t\right)q\left(t\right)-\beta r\left(t\right)$$

**Fig. 6:**
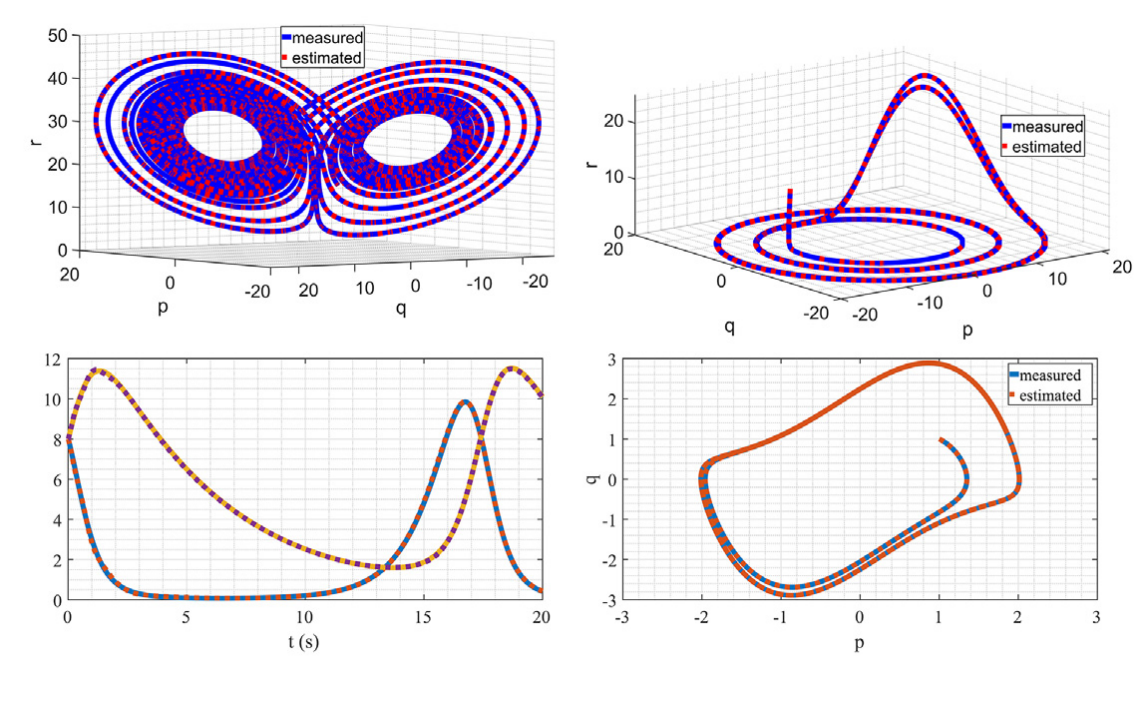
Discovering mathematical models of nonlinear dynamical systems using WyNDA.

**Fig. 7:**
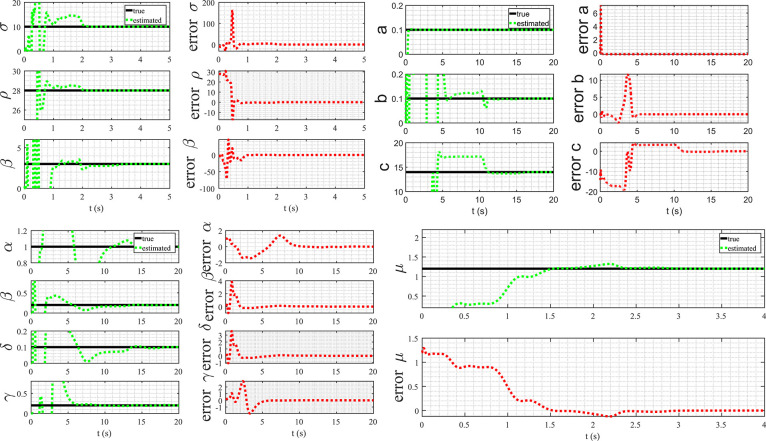
Parameter estimation of the Lorenz system.

The Lorenz system is recognized as a benchmark system in chaotic dynamics due to its remarkable sensitivity to initial conditions and the manifestation of the strange attractor phenomenon. In this example, the true value of parameters are: σ=10, ρ=28, and β=3. $y_{1}\left(k\right)$, $y_{2}\left(k\right)$, and $y_{3}\left(k\right)$ are denoted as the measurements from the sensor system, which were taken every 1 ms. The basis function for this problem is selected as follows:

(48) $$\begin{array}{c} \Phi_{j}\left(y\left(k\right),u\left(k\right)\right)={\left(\begin{array}{c} 1\\ y_{i}\left(k\right)\\ \vdots \\ y_{i}^{2}\left(k\right)\\ \vdots \\ y_{i}\left(k\right)y_{l}\left(k\right) \end{array}\right)}^{⊺}\in R^{1\times 10} \end{array}$$

for (i,l=1,2,3). The simulation results for state estimation are presented in Fig. 6 a. The initial condition of the system was ${\left(\begin{array}{ccc} -8 & 7 & 27 \end{array}\right)}^{⊺}$. It is evident from the plot that the method converges rapidly to the actual state. Note that in this example the basis function consists of 10 entries, corresponding to 30 parameters represented by $\theta_{1},\cdots ,\theta_{30}$. Specifically, the parameters σ are associated with $\theta_{2}$ and $\theta_{3}$, the parameter ρ is associated with $\theta_{12}$, and the parameter β is associated with $\theta_{24}$. The estimation results are depicted in Fig. 7. It is evident from the graph that the estimates converge to the actual values after approximately 2 s, equivalent to 2000 sampling points. The Lorenz system serves as an excellent illustration for the proposed method, given that the states exhibit high excitation for certain initial conditions. This heightened state of excitation is a key factor enabling WyNDA to accurately and swiftly discover the model.

In the second example, the Rössler attractor is considered. The Rössler attractor characterizes a continuous-time dynamical system that demonstrates chaotic dynamics, attributed to the fractal properties inherent in the attractor. The model is given by the following three dimensional differential equations:

(49) $$\dot{p}\left(t\right)=-q\left(t\right)-r\left(t\right)$$

(50) $$\dot{q}\left(t\right)=p\left(t\right)+aq\left(t\right)$$

(51) $$\dot{r}\left(t\right)=b+r\left(t\right)\left(p\left(t\right)-c\right)$$

**Fig. 8:**
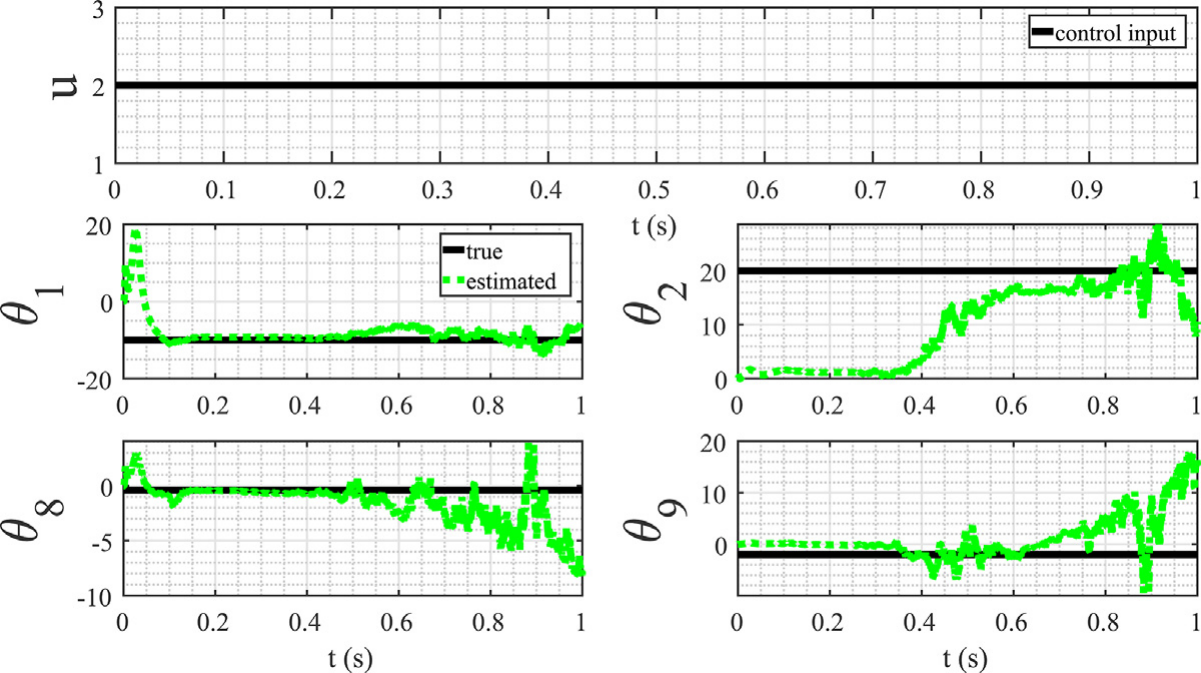
Parameter estimation of the Rössler attractor.

The model consists of three parameters, a, b, and c. In this example, a=0.1, b=0.1, and c=14 are used. In contrast to the Lorenz system, the Rössler attractor does not exhibit high excitation. Consequently, the algorithm converges at a slower pace and necessitates a greater number of data points. The diminished excitation in the Rössler attractor poses a challenge for the algorithm, as it requires a more extended observation period to accurately capture and converge to the underlying dynamics. This highlights the system-specific considerations and challenges associated with employing the algorithm across different dynamical systems. The approximation function for this problem is selected as follows:

(52) $$\begin{array}{c} \Psi \left(y\left(k\right),u\left(k\right)\right)=\left(\begin{array}{ccccc} y_{1}\left(k\right) & y_{2}\left(k\right) & y_{3}\left(k\right) & 0_{1\times 13}\\ 0_{1\times 3} & y_{1}^{2}\left(k\right) & y_{2}^{2}\left(k\right) & y_{3}^{2}\left(k\right) & 0_{1\times 10}\\ 0_{1\times 6} & 1 & y_{i}\left(k\right) & y_{i}^{2}\left(k\right) & y_{i}\left(k\right)y_{l}\left(k\right) \end{array}\right)\in R^{3\times 16} \end{array}$$

for (i,l=1,2,3). The estimation results are depicted in Figs. 6 b and 8. In this visualization, the algorithm achieves convergence after 11 s, equivalent to 11000 data points.

In the third example, the Lotka–Volterra equations is considered. The equations represent a set of first-order nonlinear differential equations commonly employed to characterize the dynamics of biological systems involving interaction between two species, one acting as a predator and the other as prey. The model has four parameters and is given by:

(53) $$\dot{p}\left(t\right)=\alpha p\left(t\right)-\beta p\left(t\right)q\left(t\right)$$

(54) $$\dot{q}\left(t\right)=\delta p\left(t\right)q\left(t\right)-\gamma q\left(t\right)$$

**Fig. 9:**
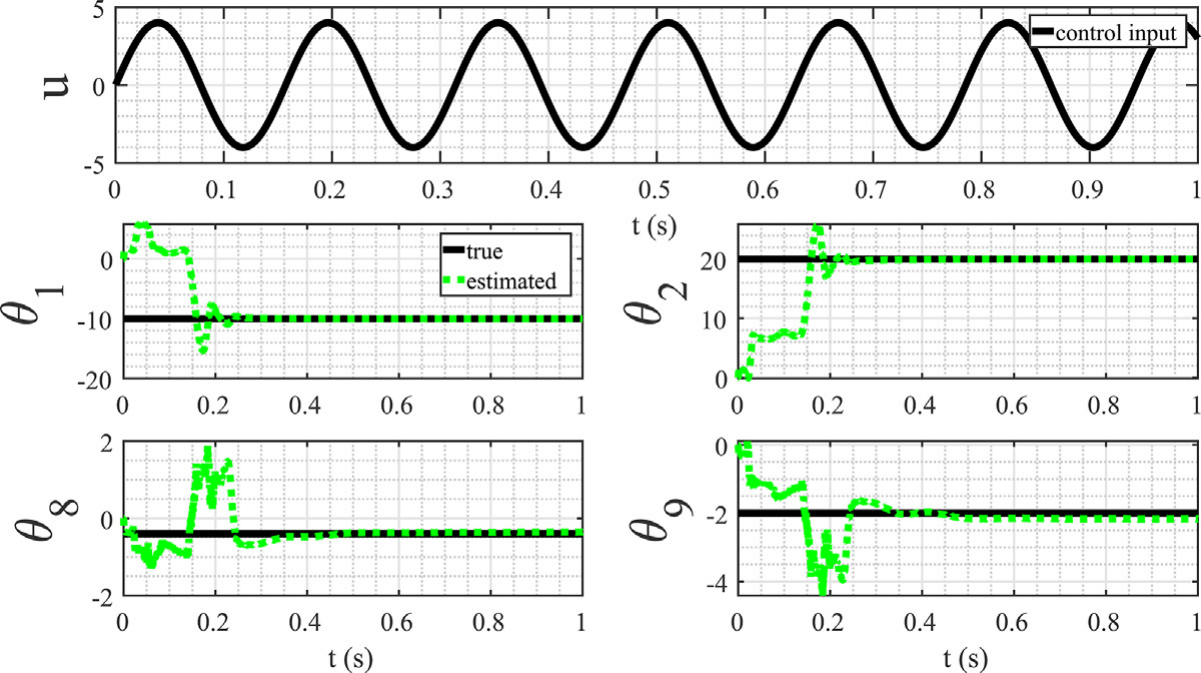
Parameter estimation of the Lotka–Volterra equations.

For the simulation, α=1, β=0.2, δ=0.1, and γ=0.2 are used. The sampling rate is set at 100 Hz. As illustrated in Fig. 9, it is evident that the estimation converges to the actual values within a time frame of 14 s, equivalent to 1400 data points. This observation underscores the efficiency of the estimation process, indicating that the algorithm accurately approximates the desired values within a specific time window and data point resolution.

The last example is the Van der Pol oscillator. The model is an oscillating system with non-linear damping. The model is given by:

(55) $$\begin{array}{ccc} \dot{p}\left(t\right) & = & q(t) \end{array}$$

(56) $$\begin{array}{ccc} \dot{q}\left(t\right) & = & \mu (1-p{\left(t\right)}^{2})q\left(t\right)-p\left(t\right) \end{array}$$

**Fig. 10:**
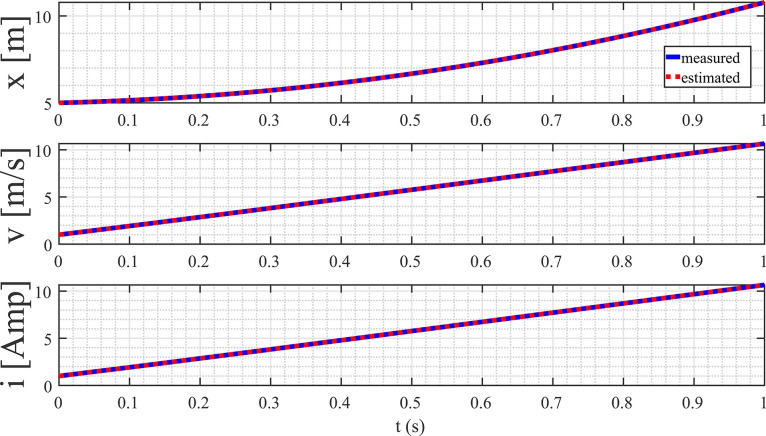
Parameter estimation of the Van der Pol oscillator.

This model features a sole parameter, denoted as μ. Given the inclusion of the term $p{\left(t\right)}^{2}q\left(t\right)$ in the model, it is judicious to incorporate the combination of $p{\left(t\right)}^{2}q\left(t\right)$ into the set of basis functions. In practice, the choice of basis functions can be versatile, allowing for the selection of a broad range to aptly capture the nonlinear dynamics inherent in the system. The results of the estimation are illustrated in Fig. 10, further attesting to the effectiveness of the proposed method. The inclusion of the selected basis function, $p{\left(t\right)}^{2}q\left(t\right)$, demonstrates the model’s capacity to accurately capture the underlying dynamics, showcasing the robustness and applicability of the proposed approach.

Table 3 provides a comparison between WyNDA, SINDy, and ARGOS in terms of the number of data observations required for each method to achieve 80% accuracy. Data for SYNDy and ARGOS were taken from Egan et al. [29]. The analysis reveals that WyNDA outperforms the other methods, particularly on systems characterized by high excitation, such as Lorenz system and Lotka Volterra equations. The efficiency demonstrated by WyNDA in accurately capturing system dynamics with a relatively limited number of data points highlights its effectiveness in scenarios where data availability is constrained, particularly in the context of highly excited systems. Notably, WyNDA operates without the need for an optimization package or machine learning, eliminating the necessity for a training phase. This characteristic renders WyNDA computationally more efficient compared to methods involving optimization and machine learning algorithms, such as SINDy and ARGOS. The absence of a training requirement simplifies the computational process, reducing overall complexity and resource demands. This characteristic enhances the practicality and speed of WyNDA, making it a computationally streamlined and effective approach for system identification.

**Table 3:** Comparison of the number of data used by WyNDA with SINDy and ARGOS.

System	Algorithm	n
Lorenz system	WyNDA	2000
	ARGOS	7944
	SINDy	NA
Rössler attractor	SINDy	1585
	ARGOS	1585
	WyNDA	11000
Lotka Volterra equations	WyNDA	1400
	ARGOS	1996
	SINDy	1585
Van der Pol oscillator	SINDy	795
	ARGOS	1000
	WyNDA	1200

### Example 3: control systems

The first two examples illustrate the method’s capability to accurately discover the mathematical models of dynamical systems. However, it’s essential to note that both examples represent special cases. In the first case, the state is excited by the initial condition, while in the second case, the state demonstrates chaotic solutions for the given parameters. The purpose of this example is to underscore the importance of excitation in the process of discovering mathematical models. In pursuit of this objective, a typical control system are examined. The dynamics of this system are captured by a set of equations, and it is crucial to emphasize that excitation, or an external influence, is necessary to unravel the underlying mathematical structure. The system’s response to excitation provides valuable insights into its behavior, allowing us to identify and understand its mathematical model more effectively. This example serves as a demonstration of the significance of excitations in the context of model discovery for dynamical systems. By showcasing the necessity of external influences for accurate modeling, a fundamental aspect that contributes to the robustness and applicability of the proposed method is highlighted. Let us consider the following control system:

(57) $$\begin{array}{ccc} \left(\begin{array}{c} {\dot{x}}_{1}\left(t\right)\\ {\dot{x}}_{2}\left(t\right) \end{array}\right) & = & \left(\begin{array}{cc} -10 & 20\\ -0.4 & -2 \end{array}\right)\left(\begin{array}{c} x_{1}\left(t\right)\\ x_{2}\left(t\right) \end{array}\right)+\left(\begin{array}{c} 0\\ 2 \end{array}\right)u\left(t\right) \end{array}$$

In the context of discovering the control system represented by (57) using measurement data of $y_{1}\left(k\right)$ and $y_{2}\left(k\right)$, a set of basis functions is employed:

(58) $$\begin{array}{c} \Phi_{j}\left(y\left(k\right),u\left(k\right)\right)={\left(\begin{array}{c} y_{1}\left(k\right)\\ y_{2}\left(k\right)\\ y_{1}^{2}\left(k\right)\\ y_{2}^{2}\left(k\right)\\ y_{1}\left(k\right)y_{2}\left(k\right)\\ u(k)\\ u^{2}\left(k\right) \end{array}\right)}^{⊺} \end{array}$$

**Fig. 11:**
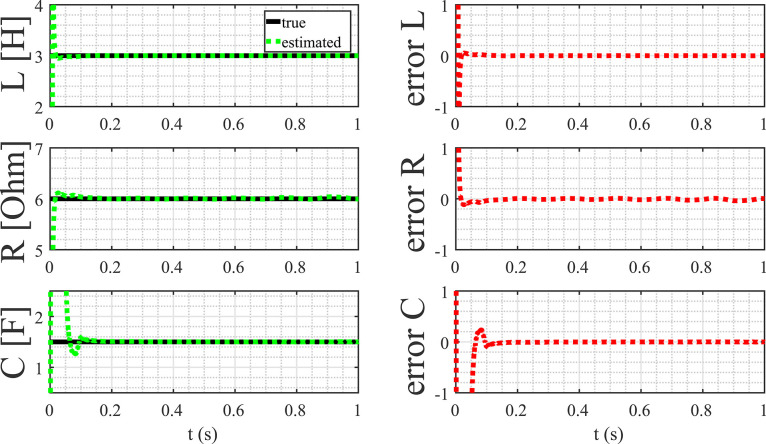
Parameter estimation with constant control input.

If the control input u(k) remains constant, the estimation results fail to converge to the actual values, as depicted in Fig. 11. This lack of convergence stems from insufficient excitation of the system states. In dynamic system identification, excitation is essential for obtaining accurate and reliable parameter estimates. When the system experiences variations and changes induced by varying inputs, it allows for a more comprehensive exploration of the state space, enabling the discovery of underlying dynamics. In this specific example, the constant nature of the control input restricts the system’s response, limiting the information available for parameter estimation. Consequently, the adaptive observer algorithm struggles to converge to the actual parameter values. This emphasizes the importance of dynamic excitation in system identification processes, as it ensures a thorough exploration of the system’s behavior and facilitates accurate parameter estimation, ultimately enhancing the reliability of the discovered mathematical models.

**Fig. 12:**
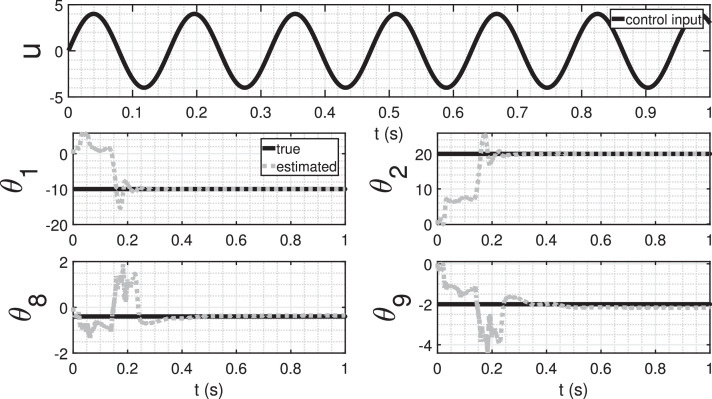
Parameter estimation with sinusoidal control input.

Fig. 12 illustrates the parameter estimation results when the input is a sinusoidal function. In this case, a convergence of the parameters to their actual values, indicating the successful discovery of the mathematical model represented by (57) was observed. This experiment highlights the crucial role of the persistency of excitation condition for the accurate identification of system dynamics. The sinusoidal input, with its varying amplitude and frequency, provides the necessary excitation to explore different regions of the state space. As a result, the adaptive observer algorithm effectively captures the system’s response to these variations, facilitating the convergence of parameter estimates. The contrast between the outcomes with constant and sinusoidal inputs underscores the significance of dynamic excitation in system identification processes. Ensuring persistency of excitation conditions enhances the robustness and reliability of discovered mathematical models, contributing to a more accurate representation of the underlying system dynamics.

### Example 4: parameter estimation

In numerous engineering applications, parameter estimation assumes a pivotal role, serving as a fundamental tool for comprehending the intricacies of a system. Whether employed for condition monitoring or predictive maintenance, accurate parameter estimation is essential for gaining insights into system behavior, facilitating informed decision-making, and enhancing the overall efficiency and reliability of engineering processes. The proposed method exhibits versatility in handling parameter estimation problems, as demonstrated through its application to a magnetic levitation model. The system under consideration can be described by the following equations:

(59) $$\begin{array}{c} \left(\begin{array}{c} \dot{x}\left(t\right)\\ \dot{v}\left(t\right)\\ \dot{i}\left(t\right) \end{array}\right)=\left(\begin{array}{c} v(t)\\ g-\frac{C}{m}{\left(\frac{i(t)}{x(t)}\right)}^{2}\\ -\frac{R}{L}i\left(t\right)+\frac{1}{L}u\left(t\right) \end{array}\right) \end{array}$$

The objective is to estimate the value of C, L, and R. Given our prior knowledge of the dynamical system’s structure, the approximation function is constructed as follows:

(60) $$\begin{array}{c} \Psi \left(y\left(k\right),u\left(k\right)\right)={\left(\begin{array}{ccc} y_{2}\left(k\right) & 0 & 0\\ 0 & 1 & 0\\ 0 & {\left(\frac{y_{3}\left(k\right)}{y_{2}\left(k\right)}\right)}^{2} & 0\\ 0 & 0 & y_{3}\left(k\right)\\ 0 & 0 & u(k) \end{array}\right)}^{⊺} \end{array}$$

The parameter vector is defined as follows:

(61) $$\begin{array}{c} \theta ={\left(\begin{array}{ccccc} \theta_{1} & \theta_{2} & \theta_{3} & \theta_{4} & \theta_{5} \end{array}\right)}^{⊺} \end{array}$$

**Fig. 13:**
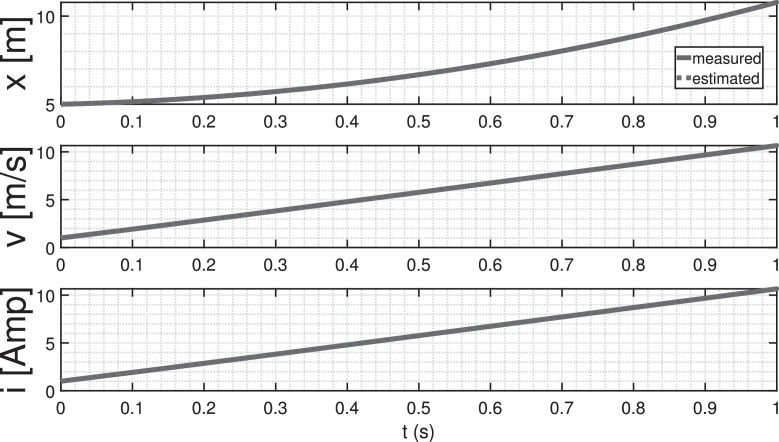
State estimation of the magnetic levitation model.

The estimation of L is derived from the estimate of $\theta_{5}$, the estimation of R is derived from the estimate of $\theta_{4}$, and the estimation of C is derived from the estimate of $\theta_{3}$. The outcomes of the state estimation process are depicted in Fig. 13. In this specific scenario, the initial state is set to ${\left(\begin{array}{ccc} 5 & 1 & 1 \end{array}\right)}^{⊺}$, with corresponding initial estimates initialized at ${\left(\begin{array}{ccc} 0 & 0 & 0 \end{array}\right)}^{⊺}$. A noteworthy observation is the rapid convergence of the estimates towards the actual state, achieving accuracy within a time frame of less than 1 ms. This compelling result serves as a tangible demonstration of the practicality and efficacy of the proposed method, particularly in the context of real-time applications.

**Fig. 14:**
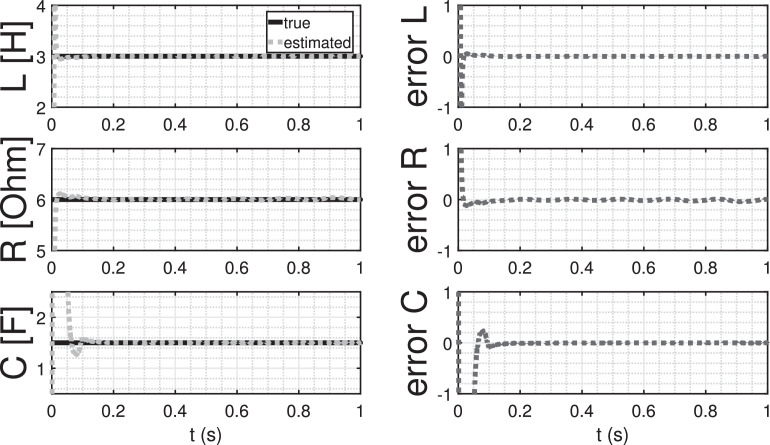
Parameter estimation of the magnetic levitation model.

The comparison of true parameter values and their corresponding estimates is illustrated in Table 4, revealing a high degree of accuracy. Moreover, an insightful observation from Fig. 14 highlights that the convergence of parameter estimates is achieved in a remarkably short span of less than 0.2 s. This swift convergence further underscores the robustness and efficiency of the proposed method in capturing accurate parameter values in a timely manner.

**Table 4:** Comparison between the true and estimated parameters.

parameter	true	estimated
L	3	2.9987
R	6	5.9939
C	1.5	1.508

## Conclusions

In this paper, an innovative methodology for discovering mathematical models of dynamical systems from data based on Wide-Array of Nonlinear Dynamics Approximation, presenting a departure from conventional approaches reliant on machine learning or optimization, has been presented. An essential feature of the proposed method is its ability to eliminate the necessity for data training, resulting in substantial computational time savings. This characteristic makes the proposed method particularly advantageous in scenarios where efficiency and speed are paramount. One notable strength of the proposed methodology lies in its robustness when handling uncertainty in measurement data. The ability to navigate and adapt to variations and imprecision in the data enhances the reliability and applicability of the approach across diverse real-world situations. This robustness contributes to the method’s potential for practical implementation, where data uncertainties are common. The numerical examples provided in the study, featuring four nonlinear mathematical models-namely, the Lorenz system, Rössler attractor, Lotka-Volterra equations, and Van der Pol oscillator-have illuminated the crucial role of persistency of excitation in the process of uncovering dynamical models. The findings emphasize that increased levels of system excitation facilitate a more rapid discovery of the model, underscoring the crucial role of dynamic input stimuli in the identification process. Notably, the main challenge and limitation for WyNDA manifest in the persistence of excitation conditions. In instances where sustained and diverse excitation signals are not consistently present, WyNDA encounters difficulty in achieving accurate and robust model estimates, hindering its effectiveness in certain operational scenarios. Therefore, ensuring a persistent excitation condition becomes a pivotal consideration for the successful application of WyNDA in system identification tasks. Moreover, the proposed method extends beyond model discovery, showcasing its applicability in the area of control and parameter estimation. The ability to identify and characterize the underlying dynamics of control systems adds an additional layer of utility to the proposed method, broadening its scope and potential applications. Additionally, the results demonstrate the effectiveness of the proposed methodology in parameter identification, emphasizing its versatility in extracting meaningful information from dynamic systems. The method’s ability to discern and estimate system parameters accurately reinforces its utility in a range of scientific and engineering applications where understanding system behavior is paramount.

## Ethics statements

The methods used in the study did not involve any human or animal subjects. No data was used or collected for this work.

## Declaration of Generative AI and AI-assisted Technologies in the Writing Process

During the preparation of this work the author used ChatGPT in order to improve readability and language. After using this tool/service, the author reviewed and edited the content as needed and takes full responsibility for the content of the publication.

## CRediT authorship contribution statement

**Agus Hasan:** Conceptualization, Methodology, Formal analysis, Writing – original draft, Writing – review & editing.

## Declaration of competing interest

The author states that he does not have any competing financial interests or personal relationships that could have influenced the work presented in this paper.

## Data Availability

No data was used for the research described in the article.
